# I-Brainer: Artificial intelligence/Internet of Things (AI/IoT)-Powered Detection of Brain Cancer

**DOI:** 10.2174/0115734056333393250117164020

**Published:** 2025-02-04

**Authors:** Abdullahi Umar Ibrahim, Ikedichukwu Onyemaucheya Nwaneri, Mercel Vubangsi, Fadi Al-Turjman

**Affiliations:** 1 Department of Biomedical Engineering, Faculty of Engineering, Near East University, Nicosia, Cyprus; 2 Research Centre for Science, Technology and Engineering (BILTEM), Near East University, Nicosia, Cyprus; 3 Department of Information Systems Engineering, Near East University, Nicosia, Mersin 10, Turkey; 4 Research Center for AI and IoT, Faculty of Engineering, University of Kyrenia, Kyrenia, Turkey; 5 Computational Materials Science Lab, Computer Science Department, HTTTC Bambili, Uinversity of Bamenda, P.O. Box 39 Bambili, Cameroon; 6 Artificial Intelligence, Software, Information Systems Engineering Departments, AI and Robotics Institute, Near East University, Nicosia, Mersin10, Turkey

**Keywords:** Artificial intelligence, Machine learning, Deep learning, Transfer learning, Computer-aided diagnosis

## Abstract

**Background/Objective:**

Brain tumor is characterized by its aggressive nature and low survival rate and therefore, it is regarded as one of the deadliest diseases. Thus, misdiagnosis or miss-classification of brain tumors can lead to miss-treatment or incorrect treatment and reduce survival chances. Therefore, there is a need to develop a technique that can identify and detect brain tumors at early stages.

**Methods:**

Here, we proposed a framework titled I-Brainer which is an Artificial Intelligence/Internet of Things (AI/IoT)-powered classification of MRI into 4 classes. We employed a Br35H+SARTAJ brain MRI dataset which contains 7023 total images including no tumor, pituitary, meningioma, and glioma. To accurately classify MRI into 4 classes, we developed the LeNet model from scratch, and implemented 2 pre-trained models which include EfficientNet and ResNet-50 as well as feature extraction of these models coupled with 2 Machine Learning (ML) classifiers namely; k-Nearest Neighbours (KNN) and Support Vector Machine (SVM).

**Results:**

Evaluation and comparison of the performance of the 3 models have shown that ResNet-50 achieved the best result in terms of AUC (99%) and ResNet-50-KNN ranked higher in terms of accuracy (94%) on the testing set.

**Conclusion:**

This framework can be harnessed by patients residing in remote areas and as a confirmatory approach for medical experts.

## 1 INTRODUCTION

The brain is a complex organ with different regions responsible for carrying out vital functions which include coordination, learning, movement, memory *etc*. Thus, malfunctioning of the brain as a result of accidents or injuries and diseases can be detrimental. Among diseases related to the brain is the brain tumor which can be defined as abnormal growth of cells in the brain and spinal cord. The symptoms of brain tumors vary according to the region of the brain impacted. However, some of the most common symptoms include persistent headaches, seizures, poor vision and hearing, nauseas, vomiting, dizziness *etc*. People at risk of brain tumor include those exposed to high doses of radiation, age, and race (meningioma are most common in black people and glioma in white people). According to cancer statistics, brain cancer is ranked as the 10th leading cause of death for men and women [1].

Brain cancer can be divided into 2, either benign or malignant or based on grades. The WHO in 2016 categorized brain tumors into 4 categories according to molecular features and histology from low to higher grades which include grades I, II, III, and IV [2]. As a result of discrepancies in diagnoses and difficulty in selecting accurate therapies based on the previous classification, 5 years later, the WHO published the 5th edition of the classification of tumors of the brain and spinal cord. The edition incorporates advances in recent knowledge of the molecular pathogenesis of brain tumors, which led to the classification of the tumor into more biologically and molecularly defined entities. Among the recent updates, glioma brain tumor are further categorized into adult- and pediatric-type [3].

Benign brain cancer is the type that grows slowly and hardly spreads. Benign brain cancer includes neuromas, meningiomas, craniopharyngiomas, *etc*. while malignant brain cancer is the type of cancer that spreads into other parts of the brain and spinal code. Types of malignant include gliomas, oligodendrogliomas, astrocytoma, glioblastomas, *etc*. Gliomas are type of brain tumor that revolves around the growth of cells that resemble glial cells. Examples of glioma include glioblastoma (which is the most common type in adults), astrocytomas, ependymomas, and oligodendrogliomas. Pineal tumors are the type of brain tumor that grows around the brain's pineal gland. Meningiomas are regarded as one of the most common types of brain tumor which can be found on the outer surface of the brain. Most meningiomas are benign. Choroid plexus which is more common in children, is another type of brain tumor that can be found in cerebrospinal fluid or fluid that surrounds the spinal cord or the brain. The embryonal tumor is the type associated with embryonal cells (cells that remain after fatal development). This type of tumor is also more common in infants [4, 5].

To diagnose suspected patients of brain tumors, medical expert relies on different approaches such as neurological examinations, laboratory assays such as biopsies, and medical imaging such as computed tomography (CT), magnetic resonance imaging (MRI), magnetic resonance spectroscopy (MRS), positron emission tomography (PET), single photon emission computed tomography (SPECT) *etc* [6]. Complete tumor screening is very challenging and time-consuming due to several steps, which include physical and neurological examinations, locating the tumor, assessing the shape, size, and location of the tumor, surgical resection through biopsy, and cell/tissue analysis for tumor grading. Biopsy is regarded as the gold standard approach for the classification, estimation of tumor grade as well as confirmation of the aggressiveness of the tumor. The procedure enables visualization of the cells and tissues and variations in terms of sizes, shapes, color, and distribution. Despite the reliance of this procedure by medical experts, the approach is tedious, time-consuming, and invasive which can be life-threatening [7, 8]. Among medical imaging techniques, MRI is regarded as one of the most reliable and accurate techniques for early identification and screening of cancer [9]. However, the use of MRI is hindered by several limitations which include difficulty in classifying or determining tumor type, error-prone, time consumption and requires an experienced radiologists for the interpretation. Accurate detection of brain tumors and discrimination between aberrant and normal tissues is highly challenging due to its wide range of traits and classes. Moreover, as a result as different variations of tumor types, MRI techniques irregularly lack apparent features that would support sound decision-making [10].

Despite progress made in the last decades, there are still challenges limiting the accurate detection of brain tumors due to their location, size, type, and shape variations. Moreover, another limitation includes the high workload faced by medical experts in terms of manual interpretation or classification of medical images. Therefore, there is a need to develop a smart and automated method for the accurate classification of brain tumors. To address these issues, scientists proposed the use of computer-aided diagnosis or detection (CADe or CADx), which applied artificial intelligence (AI)-based techniques for the screening of medical images. CADe offers several advantages, which include accurate diagnosis, consistency, and minimizing errors and workload (*i.e*., time-saving). Among different classifications of AI, machine learning (ML), and deep learning (DL) approaches are the most common techniques employed in CAD [11]. ML algorithms such as support vector machine (SVM), decision tree (DT), Naïve Bayes (NB), k-nearest neighbor (KNN), logistic regression (LR), *etc*. are employed for the classification of data through feature selection and extraction as the vital steps. In contrast to ML, DL architectures such as artificial neural networks (ANNs) and convolutional neural networks (CNNs) can extract and learn features or underlying patterns directly for the image [12].

CAD has been applied for the detection and classification of several types of cancer which include breast, skin, lungs, prostate, *etc*., from different types of medical images which include mammogram, dermatoscopic, MRI, CT scan *etc*. Accurate detection of brain tumors can be achieved through classification, segmentation and object detection. IoT-based systems are transforming many fields ranging from computer applications, robotics, military, security, architecture construction, and healthcare. The integration of IoT in healthcare or medicine is known as the Internet of Healthcare (IoHT) OR Internet of Medical Things (IoMT) [11].

One of the fields of healthcare that is benefiting from this recent technology is personalized medicine, which utilizes patient data such as signals from sensors, mobile and wearable devices, electronic health records (EHRs), social media, web-based information, *etc*. The last five years have witnessed the rise of AI/IoT-enabled frameworks for the real-time classification of diseases. AI and its subsidiaries, such as ML and DL have been applied to acquired medical data for detection or classification, prediction, and interpretation [13]. Some of the most popular examples of these frameworks include IoT-based devices developed for the detection of COVID-19 [14], skin diseases including skin cancer [15], breast cancer [16], ophthalmological disorders [17], Alzheimer’s disease [18], Parkinson disease [19]. Integration of IoT-based framework has been reported to improve medical diagnosis, monitoring disease progression, surveillance, and tracking the spread of disease [20, 21].

### 1.1 Research Questions

The formulation of precise and incisive research questions is imperative in guiding the systematic inquiry into the application of DL methodologies for the diagnosis of brain cancer. The following inquiries have been meticulously crafted to elucidate the key aspects of the study:

1. What is the discriminative efficacy of LeNet, EfficientNet, and ResNet in the classification of brain cancer, and how does their performance compare in terms of accuracy, precision, recall, F1 score, and AUC metrics?
2. To what extent does transfer learning, specifically leveraging pre-trained weights from the ImageNet dataset, enhance the performance of EfficientNet and ResNet in classifying brain cancer using the Br35H+SARTAJ brain MRI dataset?
3. How does the combination of deep learning models (LeNet, EfficientNet, and ResNet) as feature extractors with traditional classifiers (SVM and KNN) influence the overall classification accuracy, precision, recall, F1 score, and AUC for brain cancer cases?
4. What ethical considerations, regulatory frameworks, and interpretability challenges emerge in the integration of deep learning models into clinical workflows for cancer diagnosis, and how can these be navigated to ensure responsible and transparent use in healthcare settings?

### 1.2 Motivation

Several studies in the literature have attempted to classify MRI images into different classes of brain tumors such as benign and malignant or subtypes, such as pituitary, meningioma, gliomas, *etc*. using a wide variety of DL models. Although the majority of the models reported in the literature which include ResNet, AlexNet, VGGNets, Inception, *etc*. have achieved significant results in terms of accuracy and other metrics. These reported models are only implemented on a computer and are not freely accessible by patients and healthcare experts. To address this issue, few studies have reported the development of IoT-enabled frameworks that allow free access to users. Therefore, to address the limitation of the majority of research in the literature, we proposed a framework titled I-Brainer which is an artificial intelligence/internet of things (AI/IoT)-powered classification of MRI. The overall process is designed according to 4 steps. Step 1 includes data collection, pre-processing, and data split. Step 2 revolves around training and testing (a) pre-trained LeNet, ResNet-50, and EfficientNet (b) feature extraction of LeNet, ResNet-50, and EfficientNet coupled with SVM and KNN. Step 4 revolves around performance evaluation. Thus, the main contribution of this work includes:

- Developing a model from scratch for the multiclass classification of MRI into glioma, meningioma, pituitary, and no tumor.
- Implementation of 2 pre-trained models for the multiclass classification of MRI into glioma, meningioma, pituitary, and no tumor.
- Performance evaluation and comparison between a CNN developed from scratch and pre-trained models.
- Realistic comparison between highest highest-performing model and existing studies.
- Construction of AI/IoT-powered platform for online and real-time classification of brain tumors.

The rest of this work is organized as follows: Materials and method in section 2. Experimental setup up which includes data collection, pre-processing, development of model from scratch, training, and testing are discussed in section 3. The performance evaluation of the models, IoT platform, and discussion are presented in section 4 and finally, the article is concluded in section 5

## 2 RELATED WORK

The literature is gushing with studies on the application of AI and its subsidiaries which include ML and DL for the detection of diseases and abnormalities from medical images. Most of this technique revolves around feature extraction, segmentation of regions of interest (ROIs), object detection, and direct classification. Several ML and DL models have been proposed which include models developed from scratch (*i.e*., untrained) and pre-trained models based on transfer learning, hybrid models, and ensemble models.

### 2.1 AI-based Techniques for Detection and Classification of Brain Tumor

The study reported by Amin *et al*. [22] proposed extraction of deep features from inceptionv3 architecture in which score vector is acquired from SoftMax and supplied to the quantum variational classifier (QVR) for the quaternary classification of MRI images into no tumor, pituitary tumor, meningioma, and glioma. Moreover, the classified tumor was fed into Seg-network in which the actual infected region is segmented to assess the level of severity. The proposed approach was evaluated using local datasets (800 tumors/non-tumor slices of 20 patients), 2020-brats which are MRI images obtained from 259 patients of MRI (in each patient has 155 MRI slices leading 40,145 slices), with 76 cases of low glioma grades and 76 high glioma grades, the cancer genome atlas (TCGA) which contain 101 cases and multiclass dataset downloaded from Kaggle which contain 3264 (926 glioma, 937 meningioma, 500 no tumor and 901 pituitary tumors. evaluation of the proposed modified Seg-network resulted in 99.7% on the brats-2020 challenge dataset and 98.2% on the Kaggle dataset.

Biswas *et al*. [23] proposed a ternary classification of brain tumors (meningioma, glioma, and pituitary) using a hybrid approach. The study utilized MRI images obtained from figshare website which contain 563 total MRI images (246 glioma, 243 pituitary and 74 meningiomas). The overall process revolves around 4 steps which include pre-processing *via* resizing, sharpening filter, and contrast enhancement, followed by k-means clustering and feature extraction based on 2d discrete wavelet transform (DWT)and the use of principal component analysis (PCA) for features quantity reduction and finally classification using artificial neural networks (ANN). The evaluation of the proposed approach based on the Levenberg-Marquardt training function for the construction of the network resulted in an accuracy of 95.4%, the sensitivity of 94.58% and specificity of 97.83%.

To address the issue of miss-diagnosis of brain tumors, Alsubai *et al*. [10] proposed the implementation of a hybrid model which combines CNN and long short-term memory (LSTM) for accurate classification and prediction of brain tumors from MRIs. the experimental setup is designed based on data curation of MRI images from the MRI brain tumor dataset which is publicly accessible on Kaggle with 253 images (155 tumors and 98 no tumor), followed by pre-processing through cropping to remove noise, resizing and feature extraction using CNN. The performance evaluation of the proposed CNN-LSTM resulted in an accuracy, precision, recall, and f1-measure of 99.1%, 98.8%, 98.9%, and 99.0% respectively.

Tandel *et al*. [24] proposed several multiclass classifications of breast cancers from MRI. The study employed transfer learning CNN known as pre-trained AlexNet for the classification of brain tumors from MRI acquired from a publicly accessible dataset known as the repository of molecular brain neoplasia data (REMBRANDT) dataset which contains 130 tumors. To increase the number of datasets, synthetic images are generated through data augmentation (scaling, random rotation). The description of the dataset includes 47 astrocytoma, 21 oligodendroglia, 44 glioblastoma, 18 unknowns. Several classifications were conducted which include 2 classes (normal and tumorous), 3-class, 4-class, 5-class and 6-class. The proposed pre-trained CNN is compared with 6 ml classification method which includes SVM, KNN, Naive Bayes, decision tree, linear discrimination and ensemble. evaluation of the proposed model based on 10k cross-validation (k2, k5, and k10) resulted in 100%, 95.97%, 96.65%, 87.14% and 93.74% accuracies for 2, 3, 4, 5 and 6 classes.

Ismael *et al*. [25] proposed the use of DL architectures for the classification of brain cancer from MRI images. The study employed resnet-50 for the multi-classification of MRI images curated from the benchmark dataset containing 3064 MRI images of 3 brain tumor types (708 meningioma, 1426 gliomas, and 930 pituitary tumors). To maximize the dataset, several data augmentation techniques were conducted *via* flipping (horizontal and vertical), rotation, zooming, shifting, shearing, brightness manipulation, and ZCA whitening. The images were further split into 80-20% for training and testing. Evaluation of the model based on without and with augmentation resulted in 95% accuracy without augmentation and 99% accuracy with augmentation.

Sawant *et al*. [26] implemented a 5-layer CNN architecture known as LeNet for the classification of brain cancer and normal images. The model is designed with 2 convolution layers, 2 pooling layers and fully connected layer. The study employed 1800 MRI (900 tumors and 900 noncancerous) which were curated from different sources including brats, TCGA. To increase training, two data augmentation techniques which include horizontal flipping and rotation were conducted. The proposed architecture is implemented in TensorFlow and evaluated based on training and validation accuracy. Evaluation of the model based on validation accuracy resulted in 98.6%.

Ahmmed *et al*. [27] proposed the use of ml and DL frameworks for the classification of brain cancer using MRI. The study is designed according to several steps which include data acquisition, image processing, segmentation, feature extraction, detection and classification. In terms of data collection, the study acquired MRI from the oncology department of the University of Maryland Medical Center which contain 76 images (39 for the classification of normal and tumor and 37 for the classification of benign and malignant tumor stages). The pre-processing steps include conversion to jpg format, resizing and enhancement through filtration, contrast adjustment and increase of luminosity. To segment the images based on grayscale level, temper-based k-means and modified fuzzy c-means (TKFCM) clustering technique is applied. Subsequently, the first-order statistic and region property-based features are extracted from the segmented images which are further used to detect isolated tumor using SVM. Lastly, ANN is used to classify the two into benign and four malignant stages tumor. Evaluation of the proposed model based on binary classification of normal and brain tumor resulted in an accuracy of 97.37%, sensitivity of 98% and specificity of 100%.

Brindha *et al*. [28] proposed the implementation of ANN and CNN for detecting the presence of brain tumors. The overall methodology revolves around data collection in which MRI are acquired from GitHub. The dataset contains 2065 images (980 non-cancerous and 1085 brain tumors). The acquired image is processed through image resizing and partitioned into training, validation, and testing. The images are trained using 2 DL techniques. The first technique involves the use of a 7-layer ANN compiled with the Adam optimization technique and binary cross entropy loss function. The second technique revolves around the implementation of a 7-layer CNN with 5 convolution layers designed based on convolution, max pooling, and dropout filled by flattening after the 5th convolutional layer, fully connected layer, and output or classification layer. Evaluation of the model has resulted in 80.77% testing accuracy and 71.51% validation accuracy using ANN 65.21% testing accuracy and 94.00% validation accuracy using CNN.

### 2.2 AI/IoT-based Framework

El-feshawy *et al*. [29] proposed two different approaches for the detection of tumors from MRI. The first approach revolves around the use of DL-CNN directly to the acquired images while the second approach revolves around the implementation of an IoT-based framework that integrates a multiuser detection system by sending the images to the cloud for early detection of brain tumors. To train 253 MRI acquired from Kaggle titled brain MRI images for brain tumor detection dataset (with 155 tumors and 98 no tumor), a modified version of resnet-18 known as OMRES. Two vital hyper-parameters are employed to fine-tune the model by testing different optimizers using different learning rates, batch sizes as well as constant number of epochs. The second hyper-parameter revolves around assessing the impact of varying dropout rates. The modified model is compared with traditional pre-trained models and the result has shown that OMRES achieved superior performance with 98.67% highest-rated accuracy.

Lamrani *et al*. [30] proposed the use of the CNN model as an ml approach for brain tumor detection and binary classification. The study retrieved an image dataset from Kaggle repository which contain 3000 images (1500 tumor and 1500 no tumor). The images are pre-processed based on normalization of sizes and image partitioning (into 80% training, 10% validation and 10% testing). In order to train the model a CNN is implemented which is constructed based on 4 convolutional layers, 3 max-pooling layers,, flattening and 6 dense layers. The performance evaluation has shown that the model achieved 96.33% accuracy, 97.93% precision, 95% sensitivity, 75.72% specificity, and 96.44% f1-score. (Table **1**).

### 2.3 Limitation of Existing Studies

The majority of existing studies focused on the binary classification of MRI into benign and malignant as well as 3-classes. Moreover, a quite number of reported researchers proposed training MRI datasets using either a single model developed from scratch or the use of direct pre-trained models or modified versions. Consequently, most of previous studies opted for accuracy as the main metric for evaluating the performance of the model. However, in this study, we developed a 5-layer CNN architecture, the implementation of 2 pre-trained models which include EfficientNet and resnet-50 as well as feature extraction of LeNet, EfficientNet and ResNet-50. For the performance evaluation, we employed several performance metrics which include accuracy, AUC, F1-score, precision and recall. Notwithstanding, we constructed a free-accessible AI/IoT-powered platform for online and real-time classification of brain tumors.

## 3 MATERIALS AND METHODS

The experimental set up is designed based on data collection from online repository known as Kaggle, pre-processing, data split into training (80%) and testing (20%), development of 5-layer CNN (LeNet) from scratch, implementation of pre-trained models (LeNet, ResNet-50 and EfficientNet) without ML classifiers and implementation of pre-trained models with 2 classifiers which include SVM (LeNet-SVM, ResNet-50-SVM and EfficientNet-SVM) and KNN (LeNet-KNN, ResNet-50-KNN and EfficientNet-KNN) and finally performance evaluation based on accuracy, recall, precision, F1-Score and AUC. Moreover, the best performing model is deployed for real-time detection based on IoT framework. The summary of the overall methodology is illustrated in Fig. (**1**).

## 4 EXPERIMENTAL

### 4.1 Experimental Dataset

In order to train the models, we employ a publicly accessible dataset known as the Br35H+SARTAJ brain MRI dataset which contains 7023 total images which include no tumor, pituitary, meningioma and glioma as summarized in Table **2**. The dataset can be accessed through this link: https://www.kaggle.com/datasets/masoudnickparvar/brain-tumor-mri-dataset. Example of each classes is presented in Fig. (**2**).

### 4.2 Pre-processing

Normalization is the main technique employed for the pre-processing of the acquired images. These techniques play a vital role in improving the performance and generalization capabilities of ML models. The normalization technique enables uniform scaling of data across all features. Therefore, by transforming the data into a common range, normalization minimizes the influence of outliers and aids in solving issues related to variations in the magnitude and distribution of different features. Thus, we adopted the feature-wise normalization which is a widely used method in which each feature is standardized individually to have zero mean and unit variance. This approach ensures that that no single feature dominates the learning process as well as enables the ML model to capture relevant patterns and representations efficiently.

### 4.3 Data Partitioning

AI-based models are trained using a large percentage or ratio of datasets and tested using reserve (*i.e*., a small percentage of datasets). Several studies reported the use of 70:30, 75:25, and 80:20 partitioning. Thus, in this study, we opted for 80:20 to allow the models to perform very well on both training and testing sets as shown in Table **3**.

### 4.4 Deep Learning Transfer Learning

The current pre-trained models are trained using millions of images which include different animals, vehicles (such as cars, airplanes, ships *etc*.), furniture, flowers *etc*. Thus, the knowledge or weight of these trained models can be retrained to solve similar tasks with limited datasets. However, it is crucial to note that the successful implementation of these pre-trained models depends on several factors which include proper fine-tuning methods and meticulous consideration of dataset compatibility. Thus, the selection of the optimal pre-trained model as well as establishing the specific layers to be retrained requires extensive analysis and experimentation. Moreover, the selection of parameters which include learning rate and batch size plays a vital role in ensuring the effectiveness of the transfer learning process. Thus, we have adopted 2 pre-trained models which include EfficientNet and ResNet-50. Both models were selected due to their performances, high accuracy, scalability, transfer learning compatibility, and resource optimization.

### 4.5 Feature Extraction

ML models require massive amounts of datasets that demand resources to make accurate classifications and predictions. Feature extraction can be described as a process of identifying and selecting the most important features (*i.e*., information) from data. Feature extraction technique is one of the methods employed by scientists to minimize the number of resources needed and simultaneously without losing vital information. Feature extraction of images revolves around the transformation of raw data into numerical features that are compatible with ML algorithms. Among the common feature extraction approaches include dimensionality reduction, principal component analysis (PCA), linear discriminant analysis, t-distributed stochastic neighbor embedding (t-SNE) *etc*. Some of the advantages of future extraction include reducing redundant data, boosting learning speed, and improving accuracy.

As the field of medical imaging continues to grow exponentially due to the advent of cutting-edge technologies such as DL, big biomedical data (BBD), and cloud computing, the ever-increasing complexity and high dimensionality of medical data pose significant challenges. Dimensionality reduction is one of the most widely adopted approaches by scientists to address these challenges by reducing the number of features and enhancing the interpretability and efficiency of extracted features while preserving vital information. Thus, this approach is adopted in this study. After image pre-processing, features are extracted from input images and subsequently classified using either SVM or KNN.

#### 4.5.1 LeNet

The LeNet architecture was developed by Lecun and colleagues in 1989 specifically for the recognition of digits and handwritings. It is a simple CNN model with 5 main layers, which include 2 convolutional layers, 2 pooling layers, and 1 fully connected layer. Every convolution layer is designed to perform 3 operations, which include convolution (to extract spatial features), pooling (based on average pooling for downsampling) and nonlinear activation function (based on tan h).

LeNet accepts grayscale images with an input size of 32x32 which is processed by the first convolutional layer using 6 filters and 5x5 kernel size, zero padding, and 1 stride which reduces the image to a dimension of 28x28x6. The second layer, which is a pooling layer uses 6 filters, 2x2 kernel size, zero padding, and 2 stride and average pooling to downsample the output into 14x14x6 size. The second convolutional layer is characterized by 16 filters, 5x5 kernel size, zero padding, and 1 stride which reduced the image to the dimension of 10x10x16. The next layer is the second pooling layer which use a 2x2 kernel and further reduces the dimension of the image to 5x5x16. The fifth layer is a fully connected layer, where all the 400 nodes in the previous layer are connected to each of the 120 units of the fifth layer. The output layer uses SoftMax as a classifier.

#### 4.5.2 EfficientNet

Owing to its name, EfficientNet is regarded as the most efficient CNN architecture compared with previous models. Since its introduction, the model has become one of the most sought-after models for solving tasks related to image recognition and classification. EfficientNet can be described as a neural network model that uses compound scaling to achieve performance. Because traditional DL models have limitations in terms of “the better the performance, the more resources needed or consumed”. EfficientNet is designed to address these issues by increasing performance by minimizing floating point per second (FLOPs) and a number of parameters. Thus, by automatically scaling the model’s dimensions such as depth, width, and resolution, EfficientNet achieves higher accuracy.

Scaling width of DL models enables the model to capture more complex features resulting in improved accuracy. Subsequently, depth scaling enables the model to capture intricate representations of the data while scaling resolution leads to higher image resolution which provides detailed information and thus enables the model to capture underlying features. Consequently, scaling of depth, width, and resolution requires more resources, computational power, and memory. However, EfficientNet is designed to address these issues through a principled approach. The model achieved 97.3% top-5 accuracy on ImageNet dataset and 84.4% top 1 accuracy on CIFAR-100.

In terms of architecture, the model uses MBConv (Mobile Inverted Bottleneck) which can also be found in MobileNet. MBConv layers combine both inverted residual blocks and depth-wise separable convolutions. To further enhance the model, the architecture also utilizes Squeeze-and-Excitation (SE). In summary, the MBConv layer comprises depth-wise convolution, 1x1 point-wise convolution which increases the number of channels followed by another 1x1 convolution which reduces the channels back to its actual number. There are several variants of EfficientNet model which include B0, B1, B2, B3, B4, B5, B6 and B7.

#### 4.5.3 ResNet

Residual Network (ResNet) is one of the most popular CNN developed by He Kaiming. The model is developed as an improved version of VGGNet as a result of the introduction of an “identity shortcut connection” that skips 1 or more layers. Earlier on, scientists believe that “the deeper the better” which means that increasing the depth of DL architectures can increase performance. However, this was not the case, as an increasing number of layers resulted in poor performance due to the concept of vanishing gradients. The main distinction between VGGNet and ResNet is the introduction of a bottleneck as a building block which uses 1x1 convolution to minimize the number of parameters and simultaneously enables faster training. There are several variants of ResNet which include ResNet-18, ResNet-34, ResNet-50, ResNet-101, ResNet-152 and. ResNeXT. However, in this study, we implemented ResNet-50. The model is made of 50 layers in which 48 are convolutional, 1 maximum pooling layer, and 1 average pooling layer. In terms of classification, the model uses SoftMax and can classify input into 1000 categories.

### 4.6 Machine Learning Classifiers

#### 4.6.1 Support Vector Machine (SVM)

SVM is a popular supervised ML algorithm that can be used for solving both regression and classification tasks. However, like KNN, SVM is mostly deployed for solving classification problems. SVM operates based on a simple principle known as hyperplane. The algorithms operate by finding the best hyperplane that divides the data into two classes. SVM maximizes the distance or margin between data points where the closest data points are regarded as support vectors. This makes the algorithm highly efficient for binary classification. The advantages of SVM over other classification algorithms such as neural networks include high speed and better result, especially in the case of a limited number of samples. The disadvantages of running SVM include low performance in classifying noisy datasets with overlapping classes as well as less suited for training larger datasets [31].

#### 4.6.2 K-Nearest Neighbor (KNN)

KNN is ranked among one of the best ML algorithms along with SVM. It is labeled as lazy algorithm because it does not require training like other models, rather it stores that training data as a reference. KNN is a supervised ML algorithm that can be used for solving classification and prediction tasks. The ML algorithms are mostly used for classification and it operate by estimating the likelihood that a data point will become a member of one group according to the features of the group nearest to it [32]. In essence, KNN attempts to establish what group a data point belongs to by looking at the data neighboring it. Moreover, the algorithms predict by estimating the distance between the input data point and all the examples provided in the training set using Euclidean distance (*I.e*., which is a distance metric) followed by identification of k nearest neighbors to the input data point based on their distances [33].

### 4.7 Training and Fine Tuning

Feature extraction plays a crucial role in computer-aided diagnosis of skin and brain cancer. It involves extracting relevant information from medical images to enable accurate classification of cancerous and non-cancerous tissues. Consequently, hyperparameter tuning play a crucial role in the development and optimization of machine learning classifiers. In this section, we will delve into the intricacies of this process, ensuring the accuracy and effectiveness of our classifiers. By leveraging the knowledge learned from large-scale datasets, pre-trained models such as ResNet, LeNet, and EfficientNet can capture high-level features that are highly relevant for cancer diagnosis. Additionally, the feature extraction process can be further enhanced by retraining these models on more specific datasets, fine-tuning the model parameters, and freezing certain layers to prevent overfitting.

The extracted features were further classified using SVM and KNN. Consequently, SoftMax is the most common classifier used in DL models, especially in classification tasks, as it converts the model's output into probabilities across classes. Despite it benefits which include suitability for multi-class classification, computationally efficiency, and simplicity, it is not without limitations. One of the challenges of using SoftMax revolves around its susceptibility to vanishing gradient during backpropagation. SVM is another alternative robust classifier that can be incorporated after the final dense layer of DL networks in place of SoftMax. The advantages of using SVM include good generalization for small datasets in comparison with SoftMax. Moreover, SVM has better margin maximization which aids in preventing overfitting [31]. KNN on the other hand, is another classifier that can be added after the final dense layer of a neural network, instead of using SoftMax. The advantage of using KNN revolves around its non-parametric nature which makes it effective for complex or non-linear embedding as well as its post-training adaptability [32, 33].

The implementation of the KNN classifier involves a few key steps. Firstly, feature extraction is performed on the preprocessed dataset. This involves extracting the essential characteristics and patterns from the data, enabling the classifier to make informed decisions. Once the features are extracted, the KNN classifier seeks to identify the most similar training samples to a given test sample. The value of K, which represents the number of neighboring samples considered, greatly influences the outcomes of the classifier. The choice of K depends on the characteristics of the dataset. For instance, a small value of K might lead to overfitting, while a large value might introduce more noise into the classification process. Thus, hyperparameter tuning is essential to optimize the performance of the KNN classifier.

On the other hand, the implementation of the Support Vector Machines (SVM) classifier involves finding the best hyperplane that separates the samples belonging to different classes. Hyperparameter tuning is achieved by employing techniques like grid search or random search. These methods systematically explore the hyperparameter space to find the optimal combinations that yield the best classifier performance. It is important to note that hyperparameter tuning is a computationally expensive task; therefore, careful consideration must be given to the computational resources.

To achieve the best performance using classifiers, a comprehensive and rigorous hyperparameter tuning process is essential. By leveraging techniques like cross-validation, we explore various combinations of hyperparameter values systematically, aiming to identify the optimal configuration that maximizes the classifier's accuracy, precision, recall, and F1-score. This iterative process involves training and evaluating the classifiers with different hyperparameter settings on designated training and validation datasets. It enables us to find the optimal hyperparameters that strike the right balance between underfitting and overfitting.

Learning rate and batch size are two important hyper parameters that play a crucial role in training machine learning models, particularly in the context of transfer learning and cancer diagnosis. The selection of appropriate values for these hyper parameters can greatly influence the performance and convergence of the models. In this study, 1 untrained model known as LeNet and 2 pre-trained models which include ResNet-50 and EfficientNetB0 are used. The transfer learning for the pre-trained models were implemented by freezing the CNN component and training the trainable dense layers. The training was carried out using Jupiter environment using a Dell PC with NVidia GTX 1060Ti, i7 processor and 16GB RAM. The hyperparameters used for training include 0.001 learning rate and 20 epochs (Figs. **S1**-**S9**).

## 5 RESULTS

Accurate and early diagnosis of cancer is highly essential for increasing survival rate of patients and preventing immature death. The growing amount of BBD and development of several DL-based architectures and implementation of pre-trained models is changing the landscape of conventional medical diagnosis. Thus, this study is design to help radiologists and neurologists in carrying out accurate, reliable and fast diagnosis with low likelihood of miss-diagnosis. According to the outline objectives or contributions, the study developed 5-layer CNN from scratch in order to compare the performance of untrained model against pre-trained models. The second objective of the study which revolves around the use of transfer learning is addressed by implementing 2 pre-trained models which include ResNet-50 and EfficientNet. The third objective of this study is characterized by the fusion of 2 ML classifiers with DL models. This procedure is conducted in order to compare the performance of models designed with SoftMax against the ones coupled with either SVM or KNN. The next objective involved the realistic comparison between models reported in the literature review and current approaches. This objective takes into account the number of images used, the number of layers of models and pre-processing steps adopted. The last objective of this study prioritize the construction of AI/IoT-powered platform that enable real-time screening of MRI cases.

### 5.1 Performance Metrics

Performance evaluation is crucial for understanding generalizability and robustness of ML models. There are several metrics used by scientists. However, in this study we opted for 7 metrics which include accuracy, AUC, recall, precision, F1-Score, ROC and confusion matrix.

Accuracy is the most common metrics use for classification task. It measures the percentage of the correctly classified samples. It can be represented mathematically as

**Table:** 

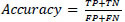	(1)

Where TP stands for true positive

TN stands for true negative

FP stands for false positive

FN stands for false negative

Accuracy can also be represented as:

**Table:** 

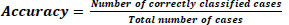	(2)

#### 5.1.1 Recall

Recall also known as “true positive rate” can be define as the portion of positive samples or cases that are correctly classified as positive. Moreover, recall can be described as the sum of positive cases that are classified as positive by the classifier. It can be represented mathematically as:

**Table:** 

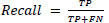	(3)

#### 5.1.2 Precision

Precision also known as “false positive rate” can be define as the portion of positive samples or cases that are incorrectly classified as positive. Moreover, precision can be described as the sum of negative cases that are classified as positive by the classifier. It can be represented mathematically as:

**Table:** 

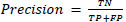	(4)

#### 5.1.3 F1-Score

F1-score is another ML performance metrics which measures model’s accuracy. The metric is mostly used when there is class imbalance. F1-score combines both recall and precision score of models using their harmonic mean. F1-score can be expressed mathematically as:

**Table:** 

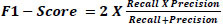	(5)

#### 5.1.4 ROC Curve

The receiver operating curve abbreviated as ROC can be expressed as the curve between TP and FP. ROC metric is very crucial in the assessment of classification approach where it displays a boundary between specificity and sensitivity.

### 5.2 Performance Evaluation of Developed LeNet

The performance evaluation of LeNet (using SoftMax as a classifier) developed from scratch resulted in 41% accuracy, 46% precision, 39% recall, 33% F1-score and 60% AUC as shown in Table **4** and summarized in Fig. (**3**).

### 5.3 Pre-trained Models without ML Classifiers

The performance evaluation of pre-trained EfficientNet resulted in 81% accuracy, 83% precision, 80% recall, 80% F1-score and 97% AUC. The performance evaluation of pre-trained ResNet-50 resulted in 86% accuracy, 89% precision, 86% recall, 86% F1-score and 99% AUC as shown in Table **4** and summarized in Fig. (**3**).

### 5.4 Feature Extraction of LeNet and Pre-trained Models Integrated with ML Classifiers

#### 5.4.1 Pre-trained Models Coupled with KNN

The performance evaluation of pre-trained Le-Net-KNN resulted in 26% accuracy, 52% precision, 26% recall, 14% F1-score and 50% AUC. The performance evaluation of pre-trained EfficientNet-KNN resulted in 87% accuracy, 87% precision, 87% recall, 87% F1-score and 95% AUC. The performance evaluation of pre-trained ResNet-50-KNN resulted in 94% accuracy, 94% precision, 94% recall, 94% F1-score and 98% AUC as shown the in Table **4**.

#### 5.4.2 Pre-trained Models Coupled with SVM

The performance evaluation of pre-trained Le-Net-SVM resulted in 30% accuracy, 31% precision, 30% recall, 16% F1-score and 52% AUC. The performance evaluation of pre-trained EfficientNet-SVM resulted in 87% accuracy, 87% precision, 87% recall, 87% F1-score and 95% AUC. The performance evaluation of pre-trained resnet-50+SVM resulted in 91% accuracy, 93% precision, 91% recall, 92% F1-score and 99% AUC as shown in Table **4**.

### 5.5 Model Design and Deployment

#### 5.5.1 Design

The design and deployment of the brain cancer detection model as a web application within the Near East University (NEU) AI-based health app is a critical phase of this project. This section delves into the comprehensive deployment methodology, emphasizing practical implementation strategies, technology choices, and integration techniques.

##### 5.5.1.1 MVC Architecture and Integration

The application adheres to the Model-View-Controller (MVC) architecture. This design choice is instrumental in segregating the application's data model (Model), the user interface (View), and the business logic (Controller), thereby promoting modular development and ease of maintenance.

##### 5.5.1.2 Seamless Module Integration

The brain cancer detection module is meticulously integrated within the NEU health app's existing ecosystem. This integration required careful planning to ensure that the new module aligns with the app’s existing functionality and user experience.

##### 5.5.1.3 Frontend Development and Design

In the case of building the user interface: The frontend is crafted using HTML, CSS, and JavaScript, prioritizing responsiveness and ease of use. The interface includes features for users to upload MRI images and view the analysis results.

##### 5.5.1.4 Backend Development and Hosting

Utilizing Streamlit for Backend: The backend is developed with Streamlit, a powerful framework that simplifies building data-intensive web applications. This choice was driven by Streamlit’s ability to handle complex data operations and integrations efficiently.

##### 5.5.1.5 Cloud Hosting on Hugging Face

Leveraging Hugging Face’s hosting solutions provides a robust and scalable cloud environment. This setup ensures the application can handle varying loads, essential for a health app with potentially high user traffic.

##### 5.5.1.6 API for Frontend-Backend Communication

The custom-designed API is pivotal for the smooth interaction between the frontend and backend. It's responsible for the secure transmission of uploaded images to the backend and relaying the predictive results back to the frontend.

##### 5.5.1.7 Data Transfer and Security

Ensuring secure and fast data transfer was paramount, given the sensitive nature of medical data. The API employs encryption and secure data handling practices to protect user privacy.

#### 5.5.2 Deployment

ResNet-50-KNN as the best performing model is employed for the development of I-Brainer (AI/IoT-based framework) which is designed to enable real-time detection of brain cancer. The system allows easy access and classification of MRI into one of the 4 classes. The detection process revolves around 3 main steps which include log in (https://health.aiiot.center/), uploading of an image, selection of diagnosis, and detection. The first step allows users to log in using a username and password. The second step involves uploading of MRI using a viable internet where the image can be pre-processed through resizing to fit the network input size. The next step involves a selection of diagnosis (*i.e*., brain cancer) and subsequently classification in a matter of seconds. The whole process (from upload to classification) can be achieved in less than a minute. The step-by-step process is illustrated in Fig. (**3**) and in a video submitted as a supplementary file.

## 6 DISCUSSION

Brain cancer is regarded as one of the deadliest diseases that are very difficult to diagnose and treat due to the intricate and complex nature of the brain. The brain biopsy procedure is regarded as the gold standard technique which revolves around a collection of tissue samples and subsequent testing using chemicals. However, this technique is life-threatening due to its invasive nature. Therefore, there is a need for non-invasive sensitive, and efficient alternatives. The use of MRI imaging has evolved as a simple alternative that allows healthcare experts to visualize the brain and discriminate between different types of benign and malignant cancer. However, this technique is influenced by several factors such as high workload which is prone to error and misinterpretation as well as inefficiency in classifying different grade of brain cancer. The application of CAD in the detection of disease offer several advantages which include improved accuracy, relief of the workload faced by healthcare experts and time-saving. The integration of CAD has been shown to address the issue of high workload and a solution in terms of ensuring accurate classification. Thus, the main objective of this study is to develop a non-invasive AI-based technique for the detection and classification of 3 brain cancers and no cancer. By retrieving the Br35H+SARTAJ brain MRI dataset which contains 7023 total images categorized into 4 classes, which include no tumor, pituitary, meningioma, and glioma, we trained (80%) and tested (20%) of the dataset according to 3 experiments. The first experiment involves the use of the LeNet model which is developed from scratch. The second experiment is characterized by the use of pre-trained models. The third experiment involves feature extraction of the developed model and pre-trained models coupled with 2 ML classifiers. Evaluation of the performance of the LeNet for the classification of brain cancer exhibited poor results across all the metrics with only AUC extended above 50% as shown in Table **4**. This can be attributed to the fact that models developed from scratch require a substantial amount of data to achieve high performance. The model is also characterized by a lack of depth, with only 5 layers compared to other models such as ResNet, DenseNet and EfficientNet. This is coherent with deeper models used in this study which include ResNet-50 and EfficientNet achieving more than 865% accuracy on reserve data. Moreover, the high performance observed in testing these models shows the strength of pre-trained models over models developed from scratch.

Deployment of AI/IoT-based frameworks in healthcare settings offers several advantages. The majority of these frameworks evolved during the COVID-19 pandemic which provide medical expert with platforms that serve as a confirmatory test or an alternative to human interpretation for the screening of COVID-19 from different types of medical images such as CT scan, X-ray, ultrasound *etc* [11]. Because screening of MRI images of patients suspected of brain cancer can be very tedious, time-consuming and prone to miss the diagnosis. Thus, the deployment of an online website embedded with AI-based models can help medical experts achieve accurate diagnoses, save time, and relieve workloads. The findings of this study coupled with the deployment of AI/IoT-based framework known as IBRAINER is now accessible to the public. The framework can be used by experts around the world to screen patients suffering from brain tumors in less than a few minutes.

### 6.1 Comparison with related Work

The study reported by [22] extracted features from InceptionV3 for 4-class classification using the 2020 BRATS dataset which comprises 40,145 images. The model achieved 99.7% accuracy, which ranked higher than our best-performing model (ResNet-50-KNN with 94%). This can be attributed to the use of a large amount of data (almost 6 times the data used to train our models). The study proposed by [22] conducted an extensive approach that combines several techniques including numerous pre-processing techniques, DWT, PCA, ANN *etc*. The hybrid approach slightly ranked higher, with 95.4% in comparison with ResNe-50-KNN [10].

In this study, the authors conducted binary classification of 253 MRI using CNN+LSTM, which resulted in higher accuracy (*i.e*., 99.1%) [10]. In this study, the authors implemented pre-trained AlexNet coupled with 6 different ML classifiers. The frameworks are trained and tested using a small dataset (130 MRI) and evaluated based on cross validation technique [24]. The best model achieved 95.97% accuracy, which is 2% higher than ResNet-50-KNN. This can be attributed to data augmentation and cross-validation in which average performance is computed.

Similarly [25], and [26] achieved 95% and 98.6% using ResNet-50 and LeNet, respectively. This can be attributed to increasing dataset using several data augmentation techniques. Hence [29], achieved 97.37% accuracy for binary classification by employing feature extraction techniques and classification using ANN. This can be attributed to the 2-class classification compared to the 4-class classification conducted in our study. The study reported by [27] trained and tested 7-layer ANN using 2065 MRI. The study reported a testing accuracy of 80.77% which ranked lower than ResNet-50-KNN [28]. achieved 98.6% using OMRESS (which is a modified ResNet-18) trained using MRI. The model is fine-tuned and optimized by adjusting several hyper-parameters [30]. applied CNN for the binary classification of brain cancer which resulted in 96.33% accuracy. Comparisons between our model and existing studies are presented in Table **5**.

The majority of the study reported accuracy as the main performance metric for ensuring model generalizability. Several studies such as [34] argued that other metrics such as AUC determine the actual performance of DL models. Moreover, the majority of ML classifiers are built through optimization of the objective function which is associated with accuracy and error rate. However, these performance metrics could be misleading for imbalanced classification. Thus, to overcome this challenge, the AUC is regarded as a desirable metric that can be employed to evaluate the models due to its robustness against class skews [34-37]. In this study, ResNet achieved 99 AUC and ResNet-50+KNN achieved 98 AUC.

### 6.2 Limitation and Future Work

Artificial intelligent models are fueled by large amounts of data, the higher the amount of the data the better the performance as long as overfitting is averted. Despite the use of 7023 MRI scan, the use of a higher amount of dataset will increase the performances of the pre-trained model. Another limitation of this study is the use of 3 DL architectures with two different classifiers, the use of several pre-trained models such as 340 VGG19, VGG16, ResNet-101, ResNet-152, Inception-ResNet DenseNet, SqueezeNet, NANSNet, *etc*. as well as hybrid and ensemble mod- 341 can improve the result. Our future research will aim to acquire more MRI and other variations such as biopsy, PET scan and SPECT scan images. Thus, training large amounts of data using best-performing models integrated with ML classifiers will enable us to develop a more robust and efficient AI/IoT-enabled framework for the classification of different types of brain cancer.

## CONCLUSION

Reliable, consistent, accurate and early detection of brain cancer is essential for correct treatment and prolonging the life of patients. Medical expert especially, pathologists, oncologists, and radiologists rely on lab bench assays and medical imaging for the accurate screening of patients suspected of brain cancer. Despite the reliance of these approaches, they are limited by several trade-offs which include a high workload in interpreting large number of cases which can lead to miss-interpretation, time consumption, the need for trained personnel *etc*. In order to address some of these challenges, scientists employ some element of industry 4.0 which revolves around the use of cutting-edge technology such as AI and its subsidiaries, IoT, cloud computing, big data *etc*. Thus, in. this study, we implemented 3 pre-trained models and a feature extraction method combined with ML classifiers for the accurate classification of brain cancer. We also developed AI/IoT framework known as I-Brainer which allows users to upload MRI images and obtain results in real-time. Despite the state of art performance achieved by ResNet-50-KNN in terms of accuracy and AUC, the study can be improved in the future by expanding the image datasets, incorporating a variety of images such as biopsy and PET scans, and implementation of hybrid and ensemble models. Therefore, this framework can be used by patients residing in remote areas and can serve as confirmatory support to medical experts such as radiologists, oncologists and neurologists.

## Figures and Tables

**Fig. (1):**
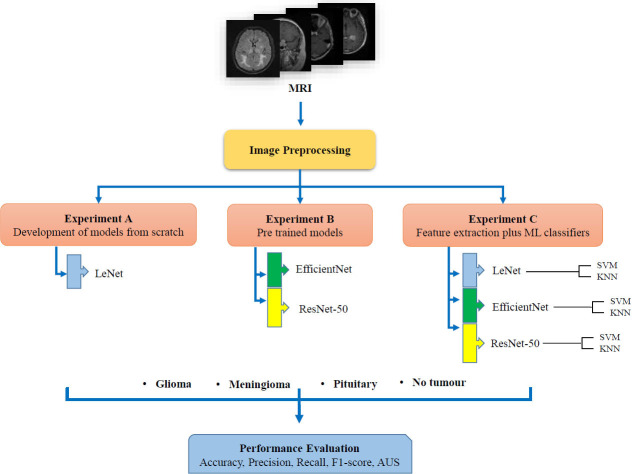
Overall methodology.

**Fig. (2):**
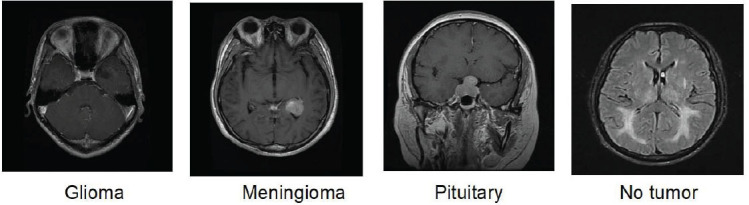
Description of dataset.

**Fig. (3):**
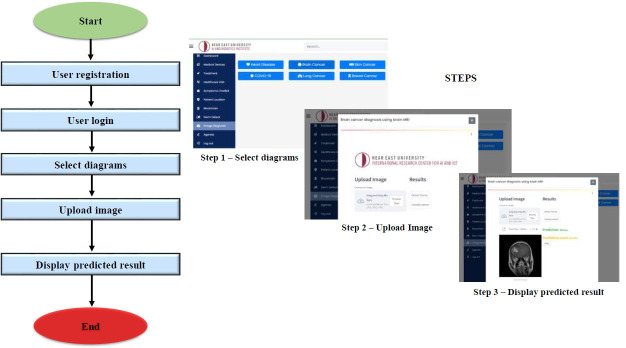
AI/IoT-based framework.

**Table 1:** Summary of related work.

**Refs.**	**Model**	**No of MRIs**	**Result (ACC/AUC)**
[22]	InceptionV3+ QVR	40,145 slices (BRATS) and 3264 images (Kaggle)	99.7% ACC using BRATS-2020 and 98.2% accuracy using Kaggle dataset
[23]	Hybrid (DWT+ANN)	563	95.4% ACC
[10]	CNN+LST	253	99.1% ACC
[24]	Pre-trained AlexNet	130	100 highest % ACC
[25]	ResNet-50	3064	99% ACC
[26]	Le-Net	1800	98.6 ACC
[27]	ANN	76	97.37 ACC
[28]	ANN	2065	80.77 ACC
[29]	OMRESS	253	98.76 ACC
[30]	CNN	3000	96.33 ACC

**Table 2:** Dataset description.

**Classes**	**Number of Images**
Glioma	1621
Meningioma	1645
Pituitary	1757
No tumor	2000

**Table 3:** Dataset description.

**Classes**	**Training (%)**	**Testing (%)**
Glioma	1297	324
Meningioma	1316	329
Pituitary	1406	351
No tumor	1600	400

**Table 4:** Performance evaluations of models trained and validated for the classification of brain cancer.

Model	**Acc (%)**	**Pr (%)**	**Rc (%)**	**F1-S (%)**	**AUC (%)**
ResNet-50	86	89	86	86	99
EfficientNet	81	83	80	80	97
LeNet	41	46	39	33	60
LeNet-KNN	30	31	30	16	52
LeNet-SVM	26	52	26	14	50
EfficientNet-SVM	89	89	89	89	97
EfficientNet-KNN	87	87	87	87	95
ResNet-50-SVM	91	93	93	92	99
ResNet-50-KNN	94	94	94	94	98

Note: * Acc: Accuracy; Pr: Precision; Rc: Recall; F1-S: F1-Score; AUC: Area Under the curve.

**Table 5:** Comparison with related work.

**Refs.**	**Model**	**No. of MRIs**	**Result (ACC %)**
[22]	InceptionV3+ QVR	40,145 slices (BRATS) and 3264 images (Kaggle)	99.7
[23]	Hybrid (DWT+ANN)	563	95.4
[10]	CNN+LST	253	99.1
[24]	Pre-trained AlexNet	130	100
[25]	ResNet-50	3064	99
[26]	LeNet	1800	98.6
[27]	ANN	76	97.37
[28]	ANN	2065	80.77
[29]	OMRESS	253	98.67
[30]	CNN	3000	96.33
This study	Pre-trained ResNet-50-KNN	7023	94

Abbreviation: * ACC: Accuracy;

## Data Availability

The data supporting the findings of the article is available in Kaggle at [https://www.kaggle.com/datasets/ masoudnickparvar/brain-tumor-mri-dataset].

## LIST OF ABBREVIATIONS

AI/IoT: Artificial intelligence/Internet of Things
PCA: Principal Component Analysis
t-SNE: t-distributed stochastic neighbor embedding
BBD: Big Biomedical Data
KNN: K-Nearest Neighbor
SVM: Support Vector Machines

## SUPPLEMENTARY MATERIALS

Supplementray material is available on the publisher's website along with the published article.
