# Supplementary material for: I-Brainer: Artificial intelligence/Internet of Things (AI/IoT)-Powered Detection of Brain Cancer
Source: Curr Med Imaging. 2025 Feb 4;21:e15734056333393. doi: 10.2174/0115734056333393250117164020 (PMC13181196; doi:10.2174/0115734056333393250117164020)
Supplement: Supplementary file 1 [file CMIM-21-E15734056333393_SD1.pdf]

1573-4056/25 *Send Orders for Reprints to reprints@benthamscience.net*

BENTHAM SCIENCE
CrossMark

Current Medical Imaging

Content list available at: https://benthamscience.com/journals/cmir

Supplementary Material

I-Brainer: Artificial intelligence/Internet of Things (AI/IoT)-Powered Detection of Brain Cancer

Abdullahi Umar Ibrahim[1,2,*], Ikedichukwu Onyemaucheya Nwaneri[3], Mercel Vubangsi[4,5] and Fadi Al-Turjman[4,6]

[1]*Department of Biomedical Engineering, Faculty of Engineering, Near East University, Nicosia, Cyprus*
[2]*Research Centre for Science, Technology and Engineering (BILTEM), Near East University, Nicosia, Cyprus*
[3]*Department of Information Systems Engineering, Near East University, Nicosia, Mersin 10, Turkey*
[4]*Research Center for AI and IoT, Faculty of Engineering, University of Kyrenia, Kyrenia, Turkey*
[5]*Computational Materials Science Lab, Computer Science Department, HTTTC Bambili, Uinversity of Bamenda, P.O. Box 39 Bambili, Cameroon*
[6]*Artificial Intelligence, Software, Information Systems Engineering Departments, AI and Robotics Institute, Near East University, Nicosia, Mersin10, Turkey*

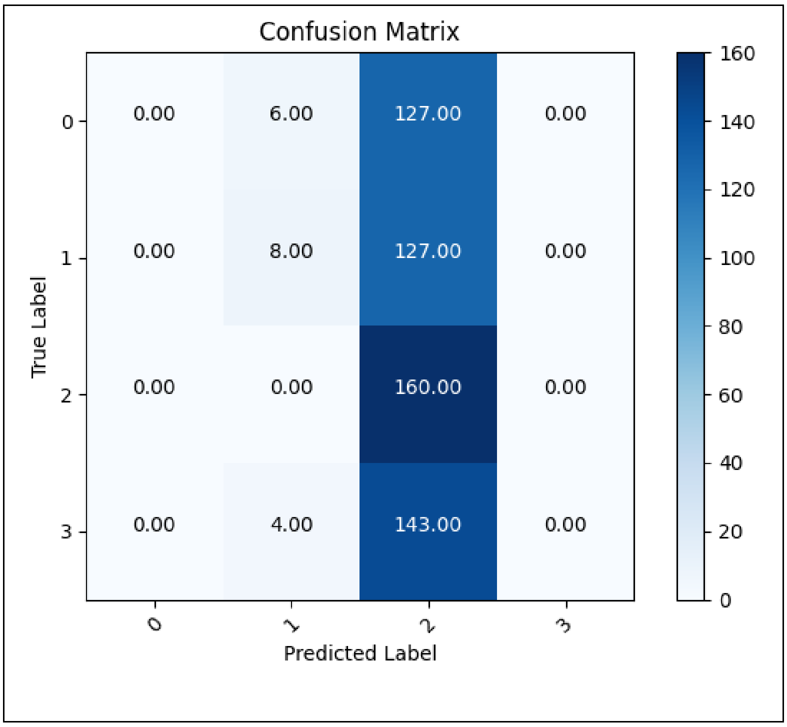

Fig. (S1). Confusion matrix depicting the training performance of lenet model on the brain MRI dataset.
The confusion matrix shows that there are 160 correct classifications for class 2, with no correct classifications for classes 0, 1, and 3. There are 415 misclassifications, with instances of all other classes being predominantly misclassified as class 2 (127 for class 0, 127 for class 1, 143 for class 3, and 6 and 8 misclassified into other classes).

DOI: 10.2174/0115734056333393250117164020, 2025, *21*, e15734056333393

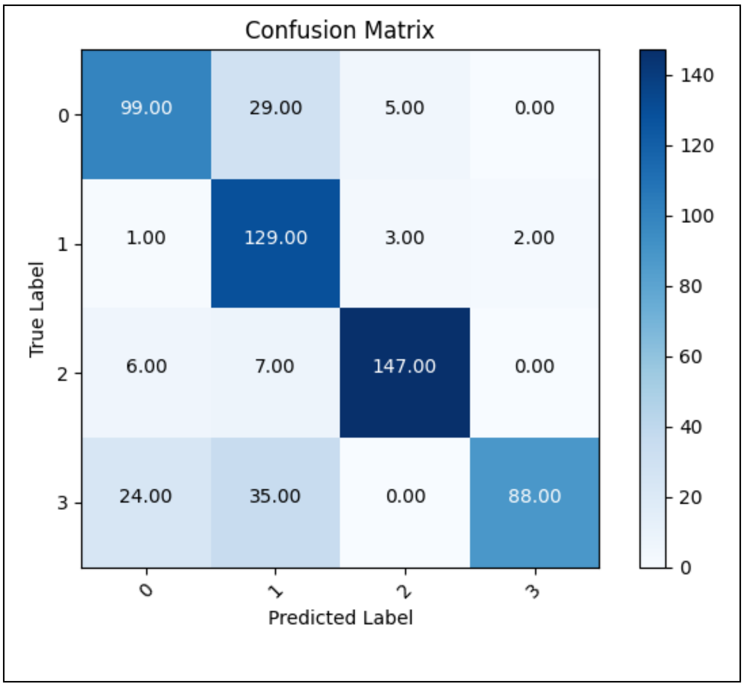

Fig. (S2). Confusion matrix depicting the training performance of the efficientnet model on the brain MRI dataset.

Fig. (**2**) illustrates the training performance of the EfficientNet model on the Brain MRI dataset through a confusion matrix. The confusion matrix on the left shows a strong performance with high correct classification counts: 99 for class 0, 129 for class 1, 147 for class 2, and 88 for class 3. Misclassifications are relatively low, with the highest misclassification being 35 instances of class 3 misclassified as class 0.

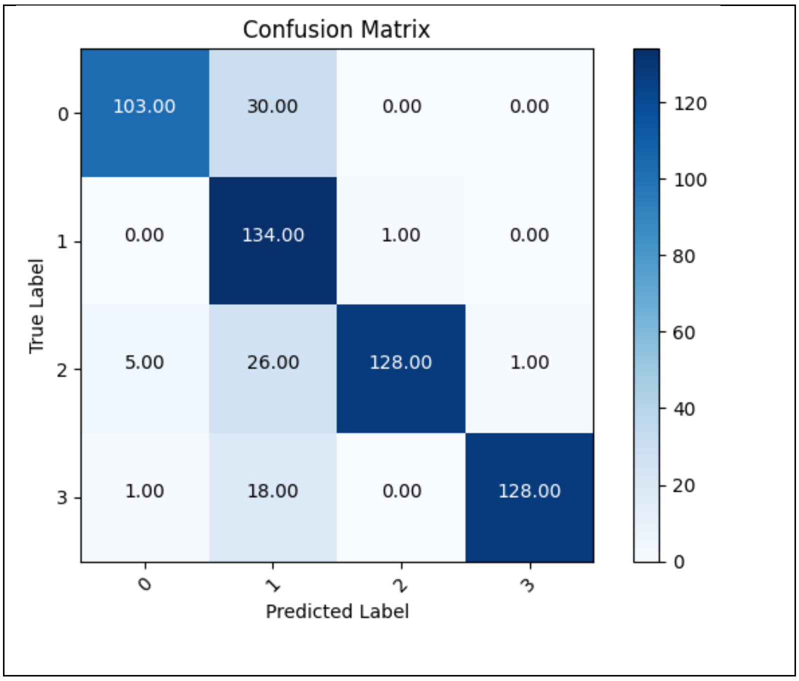

Fig. (S3). Confusion matrix depicting the training performance of resnet50 model on the brain MRI dataset.

Fig. (**3**) presents the training performance of the ResNet50 model on the Brain MRI dataset, displayed through a confusion matrix. The confusion matrix on the left highlights the model's high accuracy in classifying the different brain tumor types. Correct classifications include 103 for class 0, 134 for class 1, 128 for class 2, and 128 for class 3. Misclassifications are minimal, with the highest being 30 instances of class 0 misclassified as class 1 and 26 instances of class 1 misclassified as class 2.

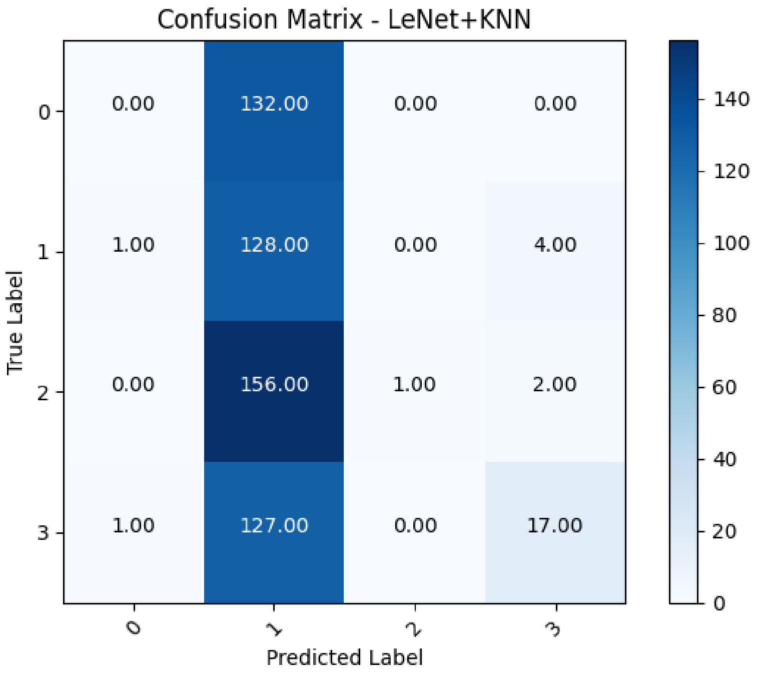

Fig. (S4). Confusion matrices depicting a comparison of the classification performance of lenet as feature extractor in combination with KNN as classifier on the tasks of brain cancer classification.

In Fig. (**4**), a detailed portrayal of the classification performance of the LeNet model acting as a feature extractor in conjunction with the KNN classifier is presented for brain cancer classification tasks. The confusion matrices reveal a notable scenario of fair classification, suggesting accurate predictions across different classes. However, the intricacies of brain cancer classification unveil a bias towards class 1, accompanied by a substantial misclassification pattern.

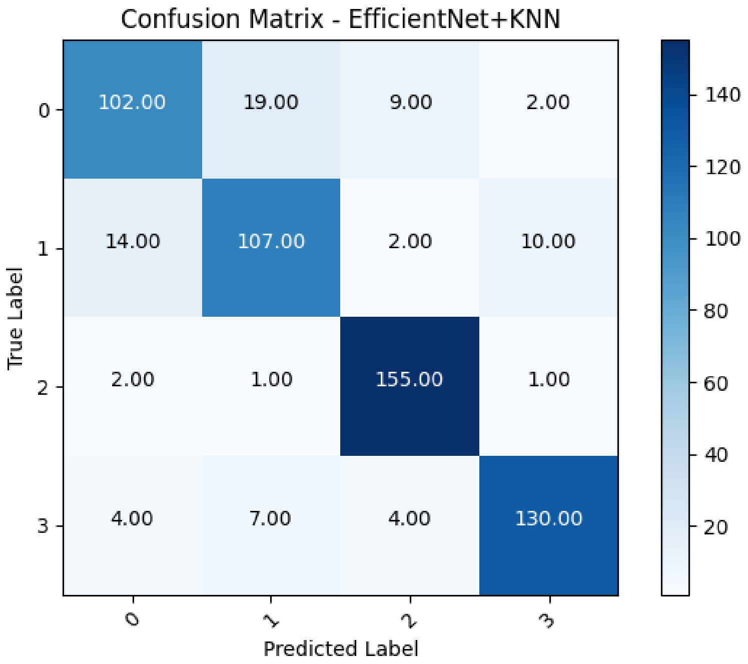

Fig. (S5). Confusion matrices depicting a comparison of the classification performance of Efficientnet as feature extractor in combination with KNN as classifier on the tasks of brain cancer classification.

In Fig. (**5**), an insightful analysis of the classification performance is presented for both skin and brain cancer using the EfficientNet model as a feature extractor combined with the KNN classifier. The confusion matrices unveil a commendable scenario of fair classification for brain cancer. The classification results exhibit fairness, suggesting that the EfficientNet model, when coupled with the KNN classifier, effectively captures the intricacies of brain cancer features without exhibiting biases towards specific classes.

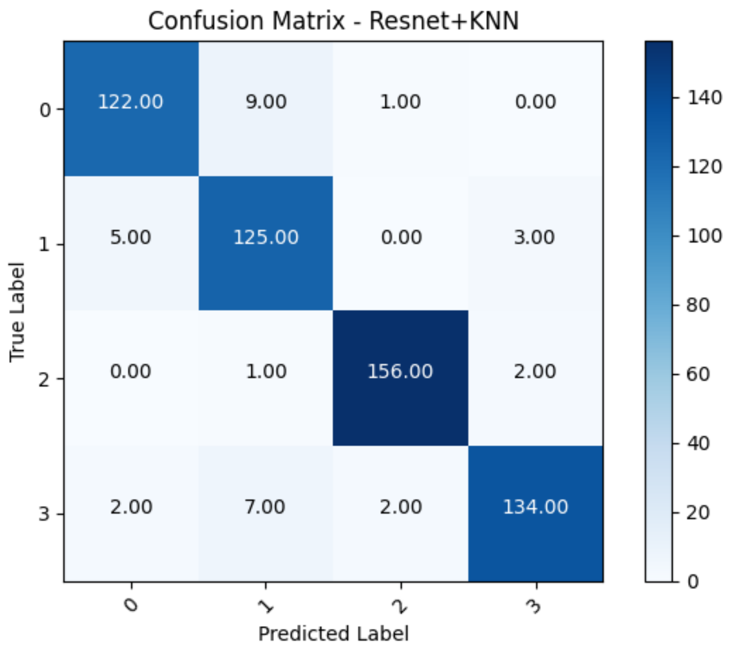

Fig. (S6). Confusion matrices depicting a comparison of the classification performance of resnet50 as feature extractor in combination with KNN as classifier on the tasks of brain cancer classification.

In Fig. (**6**), a comprehensive evaluation of the classification performance is portrayed using the ResNet50 model as a feature extractor in conjunction with the KNN classifier for brain cancer tasks. The confusion matrices illuminate an interesting pattern in the results. The brain cancer classification showcases fairly good performance with accurate predictions across different classes.

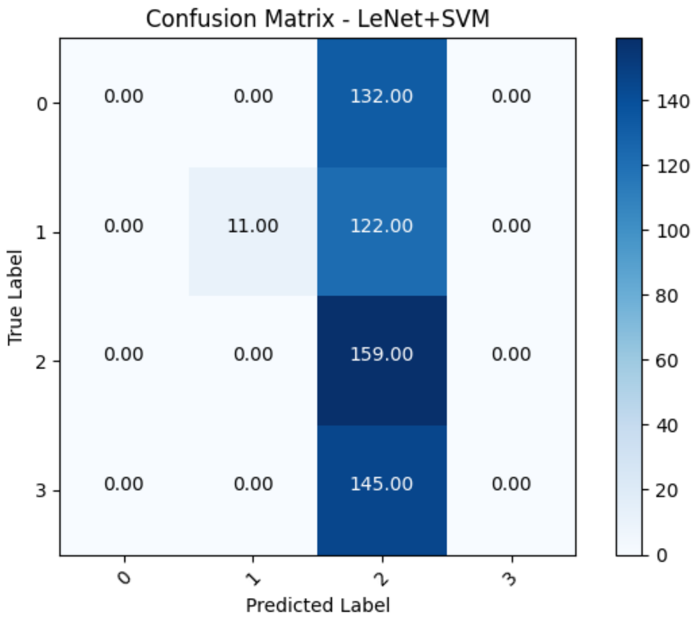

Fig. (S7). Confusion matrices depicting a comparison of the classification performance of lenet as feature extractor in combination with SVM as classifier on the tasks of brain cancer classification.

In Fig. (**7**), a compelling visual narrative unfolds through confusion matrices, providing a comparative analysis of the classification performance of the LeNet model acting as a feature extractor in conjunction with the SVM classifier. the nuanced intricacies of brain cancer classification unveil a marked of misclassification pattern with a noticeable bias towards class 2. The insights gleaned from Fig. (**7**) contribute valuable information for refining and optimizing the model's performance, guiding future enhancements and considerations in the realm of brain cancer diagnosis.

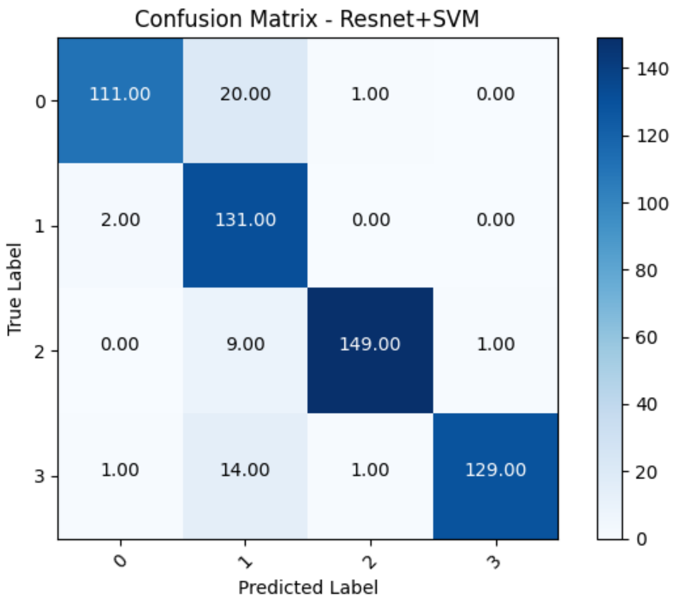

Fig. (S8). Confusion matrices depicting a comparison of the classification performance of resnet50 as feature extractor in combination with SVM as classifier on the tasks of brain cancer classification.

Fig. (**8**) presents confusion matrices illustrating the performance of a Resnet+SVM classification system for brain cancer classification. The model demonstrates a better balance between true positives and true negatives, indicating a more reliable performance.

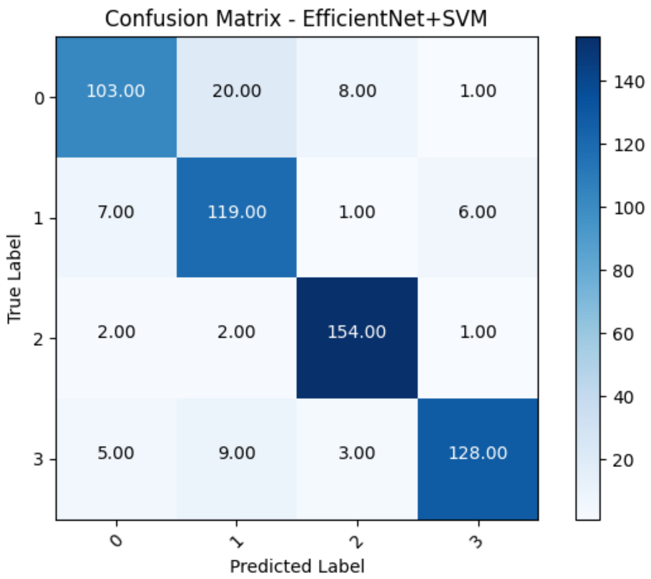

Fig. (S9). Confusion matrices depicting a comparison of the classification performance of efficientnet as feature extractor in combination with SVM as classifier on the tasks of brain cancer classification.

In Fig. (**9**), the visual narrative encapsulates the classification performance of the EfficientNet model as a feature extractor in tandem with the SVM classifier, offering insights into brain cancer classification tasks. Impressively, the confusion matrices unveil a scenario of fair classification for brain cancer. The classification performance appears well-balanced, indicating reliable predictions without a discernible bias towards any specific class. These findings affirm the model's versatility and competence in addressing diverse challenges across distinct medical imaging datasets.

© 2025 The Author(s). Published by Bentham Science Publisher.

This is an open access article distributed under the terms of the Creative Commons Attribution 4.0 International Public License (CC-BY 4.0), a copy of which is available at: https://creativecommons.org/licenses/by/4.0/legalcode. This license permits unrestricted use, distribution, and reproduction in any medium, provided the original author and source are credited.
